# Assessing the dimensionality of YFHS-Swe: a questionnaire to assess youth-friendliness in differentiated health services

**DOI:** 10.1080/16549716.2017.1380399

**Published:** 2017-10-18

**Authors:** Mazen Baroudi, Anna-Karin Waenerlund, Miguel San Sebastian, Isabel Goicolea

**Affiliations:** ^a^ Unit of Epidemiology and Global Health, Department of Public Health and Clinical Medicine, Umeå University, Umeå, Sweden

**Keywords:** Youth, friendliness, instrument, health service, psychometric analysis

## Abstract

The aim of this study was to assess the dimensionality of YFHS-Swe and identify possible unique factors in the evaluation of youth-friendliness. YFHS-Swe was answered by 1110 youths aged 16 to 25 years visiting youth clinics in Northern Sweden. Thirteen factors were identified by exploratory factor analysis and except for one factor they all proved to fit well and have good reliability when assessed by the confirmatory factor analysis. The YFHS-Swe proved to be credible and suitable for assessing youth-friendliness of differentiated health services in Sweden. With cultural and linguistic adaptations, it can be used in similar settings internationally.

## Background

Ensuring that health services are youth-friendly is important because it increases the utilization of these services and thus promotes better health behaviours.[1,2] Health systems and specific projects have used ad hoc tools and instruments to assess the youth-friendliness of health services, but to the extent of our knowledge the only validated instrument developed to measure the World Health Organization (WHO) domains of youth-friendly health services (YFHS) is YFHS WHO+ questionnaire. This questionnaire has been validated for its use in non-differentiated primary health care facilities in Bosnia-Herzegovina.[3]

**Figure 1.**
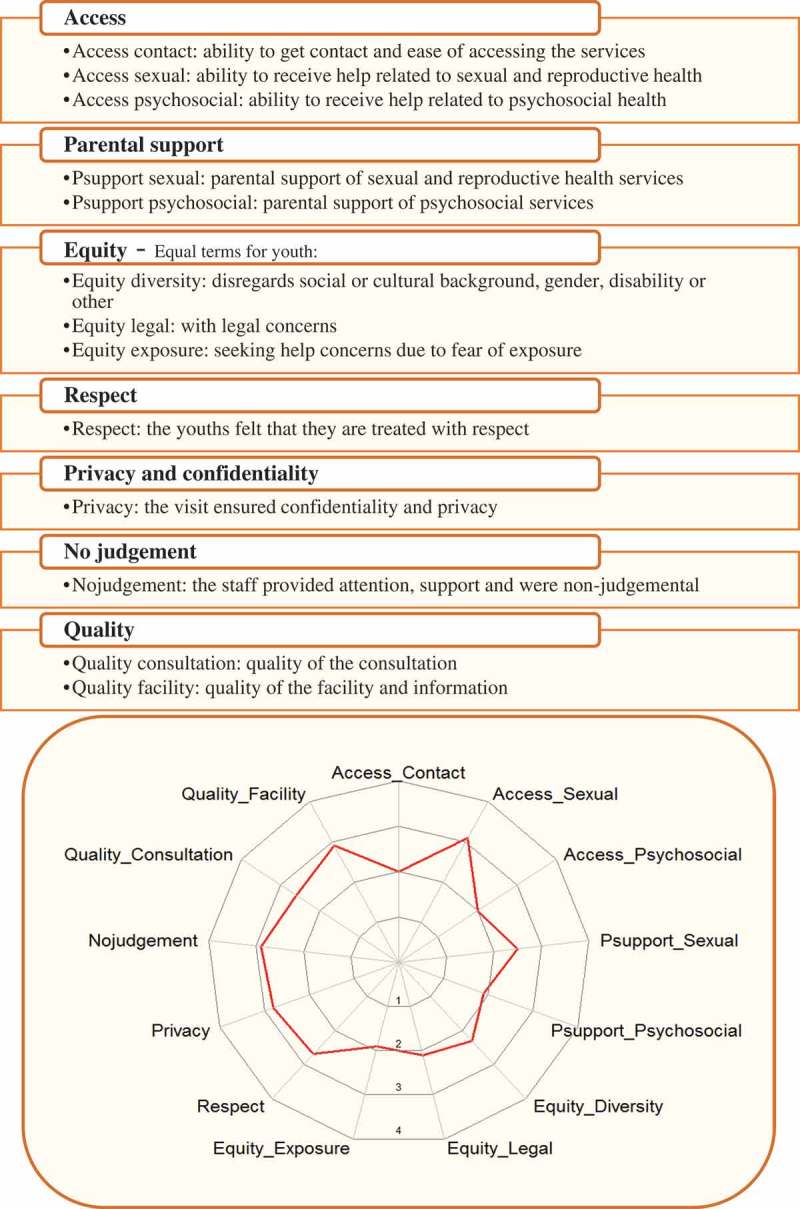
YFHS-Swe dimensions, the seven domains of YFHS-Swe and their identified factors with a representation of the factors in one of the youth clinics.

Youth aged 13–25 years accounts for around 15% of the Swedish population.[4] An increased prevalence of subjective health complaints and mental health problems have been observed recently among youth, especially in females who are twice as likely to report such problems. In addition, suicide rate, alcohol consumption and chlamydia infection have increased among this group. On the other hand smoking, pregnancy rate and abortion have decreased.[5]

Worldwide, Sweden has one of the most consolidated examples of differentiated services for youth, called youth clinics. A few youth clinics began during the early 1970s and worked mainly with health promotion. After the new abortion legislation in 1975, more clinics opened and worked on the prevention of unplanned pregnancies. Sexually transmitted infection (STI) prevention programs, especially for HIV and chlamydia, were implemented in youth clinics in the early 1980s. Later, in 1988, youth clinics were organized under the Swedish society of youth centres (FSUM), where national guidelines were developed to ensure youth-friendly health services in line with the WHO recommendations. These guidelines emphasized a holistic approach to youth and stressed the role of youth clinics as a gateway to other health services in regard to all health complaints; sexual, social, psychological or physical.[6] In order to achieve this, the minimum staff of a youth clinic should comprise at least midwife, social worker or psychologist and a doctor.[6] Currently there are around 270 clinics in the country. However, other studies have pointed out the inequality of access to these clinics and the need to reach out to disadvantaged groups, especially immigrant youth and young men.[6,7]

In order to assess the youth-friendliness of this differentiated health service, an adapted Swedish version of the YFHS WHO+ questionnaire (YFHS-Swe) was validated. YFHS-Swe proved to have good internal homogeneity and consistency over time in test–retest reliability.[8] However, factor analysis could not be performed due to the limited number of participants (74). Factor analysis is needed to: assess the dimensionality of the new instrument; identify explaining factors; ensure that each factor includes only the share meaning of its items and assure the quality of these factors.[9]

Despite the importance of youth-friendliness, the credibility of its few assessment tools are not well studied. The aim of this article was to assess the dimensionality of the YFHS-Swe to assure its quality and reliability and to identify possible factors that might be of importance for policy making. This will contribute to the credibility of this tool in assessing youth-friendliness of differentiated health services in Sweden. In addition, describing the process of the factor analysis, it might be helpful to researchers and practitioners aiming to validate similar tools.

## Methods

During the period from September 2016 to February 2017, the YFHS-Swe questionnaire was applied in 20 youth clinics in four counties in Northern Sweden. A total number of 1110 eligible participants aged 16 to 25 answered the questionnaire after their visit to the clinics.

WHO divides youth-friendliness of health services into five dimensions: accessibility; acceptability; equity; appropriateness; and effectiveness.[10] The YFHS-Swe questionnaire assesses these five dimensions through seven domains: access; parental support; equity; respect; privacy and confidentiality; no judgement; and quality. These seven domains are assessed by 85 items with a five-points Likert scale – the full questionnaire is available elsewhere.[8]

Principal component analysis (PCA) was first used to assess dimensionality and to identify the factors that explain the items in each domain. Varimax rotation was used to simplify the interpretation of the results. Factors were retained based on Kaiser’s criterion, parallel analysis of Horn [11] and thorough discussion of interpretability.

Confirmatory factor analysis (CFA) was performed to assess the quality of the factors identified by PCA. CFA also allows for each item to have its unique variance as the items cannot be entirely explained by their factors. The method of quasi maximum likelihood estimation with robust standard error (ml vce(robust)) was used, as normality could not be assumed. The method of maximum likelihood with missing value was used in privacy factor as this factor contains a question that is only answered by some of participants. To increase the validity of the models, an interaction between the error terms of the items was included when suggested by the modification indices and was conceptually reasonable. A satisfactory level of standardized root mean squared residual (SRMR) of less than 0.08 [12] and a ρ scale reliability of more than 0.7 [9] were used as the main indicators of fit and reliability. Other fit measures such as root mean square error of approximation (RMSEA) and comparative fit index (CFI) were not applicable in most of the models due to the method used in estimation.

All 13 factors were predicted from their CFA models, which allows the share meaning of items to be obtained. The predicted variables were standardized and divided into quintiles in order to visually display the factors and present them to the clinics.

## Results

Our sample consists of 90.7% females, 86.2% heterosexual and 94% Sweden-born participants. Due to the unequal access to health services among youth in Sweden, we think that this sample reflects the real users of youth clinics in Sweden.

Fourteen items were omitted to secure clearer unidimensional factors. The decision was based on statistical indications, conceptual reasoning, or indications that the youths misinterpreted the questions. (Appendix presents a list of all items, their labels and specified reasons behind the deletions).

Thirteen factors were retained from the original seven domains: ability to get contact; access to sexual and reproductive health service; access to psychosocial health services; parental support of sexual and reproductive health services; parental support of psychosocial health services; equity with diverse concerns; equity with legal concerns; fear of exposure; respect; privacy and confidentiality; no judgement; quality of consultation; and quality of facility (Figure 1). These factors could capture 61.6–83.4% of their domains variance. Eleven of the factors recorded alpha reliability of >0.80 (‘access contact’: 0.76 and ‘quality facility’: 0.53), and 10 factors reported ρ reliability of >0.80 (‘respect’: 0.78; ‘access contact’: 0.77 and ‘quality facility’: 0.58). The factors have also recorded an acceptable measure of fit (SRMR<0.08) (Table 1).

**Table 1.** YFHS-Swe factors with goodness of fit indicators and ρ reliability for each of the 13 factors of YFHS-Swe.

Domain	Factor	Related items	N	Explained variance	Alpha reliability of the scale	CFA method used	SRMR^3^	ρ reliability
Access	Access_Contact	B1-B5	1,026	12.6%	0.76	ml vce(robust)	0.05	0.77
	Access_Sexual	A4-A6	1,016	31.9%	0.89	ml vce(robust)	-	0.89
	Access_Psychosocial	A2, A3, A7-A16	844	17.1%	0.94	ml vce(robust)	0.07	0.94
Parental support	Psupport_Sexual	C4-C6	975	50.9%	0.95	ml vce(robust)	-	0.95
	Psupport_Psychosocial	C2, C3, C7-C16	939	27.2%	0.97	ml vce(robust)	0.03	0.97
Equity	Equity_Diversity	D3-D12	982	39.2%	0.96	ml vce(robust)	0.03	0.96
	Equity_Legal	D13-D15	992	15.1%	0.83	ml vce(robust)	-	0.83
	Equity_Exposure	E1-E5	982	14.8%	0.81	ml vce(robust)	0.04	0.84
Respect	Respect	F1-F3	1,003	65.6%	0.86	ml vce(robust)	-	0.87
Privacy and confidentiality	Privacy	G2, G3, G5-G7	1,009	81.7%	0.90	Mlmv	-	0.78
No judgement	Nojudgement	H1-H4	977	83.4%	0.93	ml vce(robust)	0.00	0.93
Quality	Quality_Consultation	I1-I3	954	34.5%	0.86	ml vce(robust)	-	0.86
	Quality_Facility	J1-J3	895	29.6%	0.53	ml vce(robust)	-	0.58

Abbreviations: N, number of participants; CFA, confirmatory factor analysis; SRMR, standardized root mean squared residual; ml vce(robust), method of quasi maximum likelihood estimation with robust standard error; mlmv, method of maximum likelihood with missing value.

## Discussion and conclusion

Our results suggest that the original seven domains of YFHS-Swe cannot capture all the dimensions of youth-friendliness and these 13 identified factors might be of an importance in assessing the friendliness of differentiated youth services. The robust process we followed and the large sample size of this study increase the credibility of these results.[13] During the analysis, the discussion among researchers was highly valuable in supporting our statistical findings with conceptual reasoning and in finding appropriate labels to the identified factors.

On the other hand, the low number of males and immigrants in our sample might make our results unrepresentative of the Swedish youth population. However, the aim of the instrument was to assess the perception of youth-friendliness among those who have visited the youth clinics, not among youth in general. Another limitation could be that we only surveyed the four sparsely populated northern counties, which might not represent youth clinics in other parts of Sweden. Future research could embrace a nationwide perspective including clinics from different parts of Sweden and focus on the underrepresented groups.

YFHS-Swe proved to be credible and suitable for the Swedish context. We therefore can recommend the usage of this questionnaire to assess youth-friendliness nationally and, with some cultural and linguistic adaptations, in other differentiated youth health services internationally. The identified factors might be of an importance to capture different dimensions of youth-friendliness.

## Supplementary Material

Supplementary Material

## Paper context

Ensuring youth-friendliness can increase the utilization of health services and promote better health behaviours among youth. A new instrument to assess youth-friendliness in the Swedish youth clinics was validated; however, a psychometric analysis is needed to assure its quality, reliability and goodness of fit. The questionnaire is proved to be credible and suitable and might be of importance for policy making, youth clinics and as a basis for validating other instruments to assess youth-friendliness in other countries.
