# Supplementary material for: Assessing the dimensionality of YFHS-Swe: a questionnaire to assess youth-friendliness in differentiated health services
Source: Glob Health Action. 2017 Oct 18;10(1):1380399. doi: 10.1080/16549716.2017.1380399 (PMC5678427; doi:10.1080/16549716.2017.1380399)
Supplement: Supplementary Material [file ZGHA_A_1380399_SM2042.docx]

**Appendix , List of all items in YFHS-Swe questionnaire, their labels and specified reasons behind the deletions:**

**Subscale: access**

*If you had the following problems, do you think you would be able to receive help at this clinic?*

*Grade every question from 1 (never) to 5 (always) and put a tick on the alternative that fits.*

| Label | Question | Factor identified |
| --- | --- | --- |
|  | Physical problems, for example gynaecological concerns, painful intercourse, foreskin problems, fatigue, painful or irregular menstruation | Omitted, the item conceptually is related to more than one factor of access |
|  | Mental problems, for example, worry, feeling blue, stress, depression, anxiety, thoughts about hurting yourself or suicidal thoughts | Access_Psychosocial |
|  | Questions concerning questions related to sexual orientation or gender identity | Access_Psychosocial |
|  | Concerning questions about or suspicions of pregnancy | Access_Sexual |
|  | Concerning questions about or to run a test for sexually transmitted infections, for example, Chlamydia, HIV, Herpes, Gonorrhoea | Access_Sexual |
|  | Questions about methods to avoid pregnancy (contraceptives) | Access_Sexual |
|  | Concerning relationship to friend/partner | Access_Psychosocial |
|  | Smoking or help to stop smoking cigarettes | Access_Psychosocial |
|  | Problems with alcohol | Access_Psychosocial |
|  | Problems with marijuana or other drugs | Access_Psychosocial |
|  | Problems with parents or family | Access_Psychosocial |
|  | Problems with work/unemployment, school or university | Access_Psychosocial |
|  | Questions about food, exercise or sleeping habits | Access_Psychosocial |
|  | Concerning sexual actions that have occurred against your will | Access_Psychosocial |
|  | Concerning that someone has, or tried to, hurt you, or that you did it to someone else, for example fights, hits or kicks | Access_Psychosocial |
|  | Concerning that someone made you feel bad with something they said, for example threatened or insulted you | Access_Psychosocial |

*The following questions concern waiting times and how easy it is to receive help here. Grade the following questions from 1 (very bad) to 5 (very good). Put a tick on the alternatives that fit.*

| Label | Question | Factor identified |
| --- | --- | --- |
|  | What do you think of the opening hours of this service? | Access_Contact |
|  | What do you think about the possibility of getting a booked appointment here? | Access_Contact |
|  | What do you think about the time you had to wait in the waiting room? | Access_Contact |
|  | What do you think about the possibility to be in contact, by phone, with this service? | Access_Contact |
|  | What do you think about the possibility to be in contact with the staff here (personally or by phone)? | Access_Contact |

**Subscale: parental support**

*If your parents, or another important adult in your family or close to you, knew you had this problem, would they advise you to seek help at this clinic? Grade the following questions from 1 (never) to 5 (always). Put a tick on the alternatives that fit.*

| Label | Question | Factor identified |
| --- | --- | --- |
|  | Physical problems, for example stomach ache, cough, acne, , fatigue, painful or irregular menstruation | Omitted, the item conceptually is related to more than one factor of access, better α reliability after omitting |
|  | Mental problems, for example, worry, feeling blue, stress, depression, anxiety, thoughts about hurting yourself or suicidal thoughts | Psupport_Psychosocial |
|  | Questions concerning questions related to sexual orientation or gender identity | Psupport_Psychosocial |
|  | Concerning questions around or suspicions of pregnancy | Psupport_Sexual |
|  | Concerning questions about or to run a test for sexually transmitted infections, for example, Chlamydia, HIV, Herpes, Gonorrhoea | Psupport_Sexual |
|  | Questions about methods to avoid pregnancy (contraceptives) | Psupport_Sexual |
|  | Concerning relationship to friend/partner | Psupport_Psychosocial |
|  | Smoking or help to stop smoking cigarettes | Psupport_Psychosocial |
|  | Problems with alcohol | Psupport_Psychosocial |
|  | Problems with marijuana or other drugs | Psupport_Psychosocial |
|  | Problems with parents or family | Psupport_Psychosocial |
|  | Problems with work/unemployment, school or university | Psupport_Psychosocial |
|  | Questions about food, exercise or sleeping habits | Psupport_Psychosocial |
|  | Concerning sexual actions that have occurred against your will | Psupport_Psychosocial |
|  | Concerning that someone has, or tried to, hurt you, or that you did it to someone else, for example fights, hits or kicks | Psupport_Psychosocial |
|  | Concerning that someone made you feel bad with something they said, for example threatened or insulted you | Psupport_Psychosocial |

**Subscale: equity**

*Do you believe that all groups of young people, have the possibility to receive the health care they are entitled to at this clinic? For each of one of the groups below, grade how likely that is from 1 (never) to 5 (always). Put tick on the alternatives that fit.*

| Label | Question | Factor identifies |
| --- | --- | --- |
|  | Young that are very young | Omitted, the item is open to different interpretation, better α reliability after omitting |
|  | Young that are older | Omitted, the item is open to different interpretation |
|  | Regardless of their gender identity | Equity_Diversity |
|  | Regardless of their ethnical background | Equity_Diversity |
|  | Regardless of their social background | Equity_Diversity |
|  | Regardless of their religion | Equity_Diversity |
|  | Regardless of what they wear (clothes and appearance) | Equity_Diversity |
|  | Even if they live on the street | Equity_Diversity |
|  | Because of a certain sexual orientation | Equity_Diversity |
|  | Even if they have a disability | Equity_Diversity |
|  | Even if they have mental illness | Equity_Diversity |
|  | Because they use drugs | Equity_Diversity |
|  | Even if they are violent | Equity_Legal |
|  | Even if they are involved in selling/buying sex | Equity_Legal |
|  | Even if they are in Sweden without legal permit (E.g. living hidden after declined asylum or without legal papers) | Equity_Legal |

*There might be some reasons that certain groups of young people abstain from seeking help on the service. Do you think a young person would abstain from seeking help on this service because… Grade following statements from 1 (never) to 5 (always). Put tick on the alternative that fit.*

| Label | Question | Factor identified |
| --- | --- | --- |
|  | They are afraid that their parents would find out or disapprove of it | Equity_Exposure |
|  | They are afraid that teachers, staff at school or the principal would find out | Equity_Exposure |
|  | They are afraid that the police would find out | Equity_Exposure |
|  | They are afraid that their employer would find out | Equity_Exposure |
|  | They are afraid that their friends would find out | Equity_Exposure |

**Subscale: respect**

*The following questions concern how you felt that you were treated on your visit to the youth clinic today. Grade the following questions from 1 (very bad/uncomfortable) to 5 (very good/comfortable). Put tick on the alternatives that fit.*

| Label | Question | Factor identified |
| --- | --- | --- |
|  | How were you treated by the staff that you made the visit to today? | Respect |
|  | During the time of your visit, how comfortable did you feel? | Respect |
|  | How much trust do you have in this person? | Respect |
|  | How would you grade the treatment from other staff today? | Omitted, the item is not applicable for all youths, better α reliability after omitting |

**Subscale: privacy**

*The following questions concern the confidentiality of the staff and how you were treated here today. Grade every question from 1 (very bad/never) to 5 (very good/always). Put ticks on the alternatives that fit.*

| Label | Question | Factor identified |
| --- | --- | --- |
|  | During this or on previous visits to this service, did you receive information regarding the confidentiality of the staff? | Omitted, better α reliability after omitting |
|  | How certain are you that the ones working in this service will not talk about your problems with other adults, for example your parents, if you don´t agree to it? | Privacy |
|  | How much do you trust that the staff will keep your problems confidential, i.e. so that no one else gets to know about your problems? | Privacy |
|  | Repeated question as above | Omitted, the item is repeated |
|  | Conversations with the staff are conducted in such a way that no one else can hear what you are talking about? | Privacy |
|  | Examinations are conducted in such a way that no one else can see or hear you? | Privacy |
|  | Only answer if you came to the visit together with an adult: Did the staff ask you if you wanted to be alone, without the presence of a parent or another adult? | Privacy |

**Subscale: no judgement**

*In this part you will be questioned about today’s visit to one of the persons working in this clinic. Grade statements from 1 (don´t agree) to 5 (totally agree). Put ticks on the alternatives that fit.*

| Label | Question | Factor identified |
| --- | --- | --- |
|  | The staff gave you their full attention | Nojudgement |
|  | The staff respected your opinions and decisions | Nojudgement |
|  | The staff treated you in a supporting and caring way | Nojudgement |
|  | The staff had an unprejudiced attitude towards you | Nojudgement |

**Subscale: quality**

*Think about today’s visit. Grade the statements from 1 (don´t agree) to 5 (totally agree) depending on to what extent you agree. Put ticks on the alternatives that fit.*

| Label | Question | Factor identified |
| --- | --- | --- |
|  | You received treatment or help that met your expectations | Quality_Consultation |
|  | The staff explained to you, with words you could understand | Quality_Consultation |
|  | The time that was allowed was enough for you to ask all the questions you wanted to ask | Quality_Consultation |
|  | Have you ever postponed a visit at the youth clinic/youth health because of unsuitable opening hours? | Omitted, indication that the participants misinterpreted the question (item is in reverse order) |
| Only answer the following questions if they are relevant according to your visit. If not, proceed to question | | |
|  | You understood the examination or treatment suggested by the staff | Omitted, indication that the participants misinterpreted the question (item is only applicable for some participants) |
|  | The staff asked you what treatment you would prefer | Omitted, as above |
|  | The staff explained the treatment to you and why it was suggested | Omitted, as above |
|  | The staff discussed with you the pros and cons that you could expect from the treatment | Omitted, as above |
|  | The staff explained to you what tests they were doing | Omitted, as above |
|  | The staff explained to you the results of the tests or the examinations or told you when you will get to know what the tests showed | Omitted, as above |

*Think about your visits to this clinic. Grade the questions from 1 (very bad/nothing) to 5 (very good/lots) depending on to what extent you agree. Put ticks on the alternatives that fit.*

| Label | Question | Factor identified |
| --- | --- | --- |
|  | How would you grade the waiting room and the clinic facility? | Quality_facility |
|  | Was there information material concerning youths’ health? | Quality_facility |
|  | How would you grade the quality of the information in the information material? | Quality_facility |
